# A Survey on Autism Care, Diagnosis, and Intervention Based on Mobile Apps Focusing on Usability and Software Design

**DOI:** 10.3390/s23146260

**Published:** 2023-07-09

**Authors:** Xiongyi Liu, Wenbing Zhao, Quan Qi, Xiong Luo

**Affiliations:** 1 Department of Curriculum and Foundations, Cleveland State University, Cleveland, OH 44115, USA; x.liu6@csuohio.edu; 2 Department of Electrical and Computer Engineering, Cleveland State University, Cleveland, OH 44115, USA; 3 College of Information Science and Technology, Shihezi University, Shihezi 832003, China; q.qi@ieee.org; 4 School of Computer and Communication Engineering, University of Science and Technology Beijing, Beijing 100083, China; xluo@ustb.edu.cn

**Keywords:** autism, mobile apps, smartphones, user-centered design, augmentative and alternative communication, video modeling, virtual reality, gamification, behavior change techniques, cognitive behavioral training, behavior modeling training

## Abstract

This article presents a systematic review on autism care, diagnosis, and intervention based on mobile apps running on smartphones and tablets. Here, the term “intervention” means a carefully planned set of activities with the objective of improving autism symptoms. We guide our review on related studies using five research questions. First, who benefits the most from these mobile apps? Second, what are the primary purposes of these mobile apps? Third, what mechanisms have been incorporated in these mobiles apps to improve usability? Fourth, what guidelines have been used in the design and implementation of these mobile apps? Fifth, what theories and frameworks have been used as the foundation for these mobile apps to ensure the intervention effectiveness? As can be seen from these research questions, we focus on the usability and software development of the mobile apps. Informed by the findings of these research questions, we propose a taxonomy for the mobile apps and their users. The mobile apps can be categorized into autism support apps, educational apps, teacher training apps, parental support apps, and data collection apps. The individuals with autism spectrum disorder (ASD) are the primary users of the first two categories of apps. Teachers of children with ASD are the primary users of the teacher training apps. Parents are the primary users of the parental support apps, while individuals with ASD are usually the primary users of the data collection apps and clinicians and autism researchers are the beneficiaries. Gamification, virtual reality, and autism-specific mechanisms have been used to improve the usability of the apps. User-centered design is the most popular approach for mobile app development. Augmentative and alternative communication, video modeling, and various behavior change practices have been used as the theoretical foundation for intervention efficacy.

## 1. Introduction

The development of mobile technologies has opened a new method for healthcare delivery, patient self-care, patient data collection, and health/medical research. Mobile technologies consist of increasingly powerful hardware in portable forms, such as smartphones, tablets, and smartwatches, mobile operating systems (primary Android and iOS), and various mobile apps. This new type of platform is much more accessible to the general population with the availability of lightweight and easy to carry mobile devices and intuitive mobile apps. Sometimes, additional wearable devices may be used in conjunction with mobile apps to collect physiological data (such as heart rate, body temperature, and skin resistance) or to provide visual guidance (such as Google Glasses) or tactile live feedback (such as wristbands). This line of research and practice is referred to as mobile health [1], or mHealth, which is one form of smart healthcare [2].

Mobile health has a number of advantages over traditional in-person treatment delivery [1]: (1) it is more cost-effective because the treatment may be delivered remotely; (2) it is easier to access because the patient may receive the treatment at home instead of having to commute to a clinic or hospital; and (3) it offers a new way of healthcare delivery that is not possible before, such as via virtual or augmented reality with avatars and 2D or 3D scenes, and integration with gamification mechanics, which could be more engaging and therefore more conducive to patient adherence to the prescribed interventions. Mobile health also transforms the way of collecting data regarding the patients, such as monitoring the level of physical activity using the built-in sensors of the mobile devices and the mental state via ecological momentary assessments.

In this paper, we focus on the use of mobile apps for autism care, diagnosis, and intervention. In addition to the personal interest of the authors, a big reason why we focus on the combination of autism care and mobile apps is because mobile apps could be the best platform to deliver both short-term and long-term autism care. Autism spectrum disorder (ASD) is a neurodevelopmental disorder and has no known cure at this time [3]. Unlike other physical or mental diseases or disorders, ASD is known for its heterogeneity, although the core domains of difficulties have been reliably identified: (1) lack of social skills; (2) impaired communication; and (3) unusual restricted and repetitive behaviors [3]. Some individuals with ASD are non-verbal. Many individuals with ASD tend to have an version to engaging in direct in-person conversations, but are highly proficient and comfortable in using technologies, particularly smartphones and mobile apps. This makes mobile apps an ideal platform to deliver care and intervention to individuals with ASD, and also to facilitate academic learning for young and adolescent individuals with ASD. Indeed, numerous mobile apps have been developed for the autism community.

There is a large body of literature on autism, and the subject matter has been reviewed many times throughout the last several decades since autism was first described in 1943 [4]. Despite intense research over several decades, the etiology and biology of autism are still not well understood. As such, the symptoms of autism are mitigated via educational and behavioral interventions [3]. This fact further elevates the potential value of mobiles apps as a platform to deliver autism care. As we will show in this paper, mobile apps have also been used to support the caregivers (usually parents) of the individuals with ASD, and to facilitate the collection of data regarding the symptoms of the individuals for assessment.

### Related Reviews

We are aware of several systematic reviews related to the use of mobile apps for autism study in various aspects. In [5], mobile apps are considered as one of the several technologies to assess, monitor, and treat neurodevelopmental disorders, which included both autism and attention deficit/hyperactivity disorders (ADHD). Four aspects of the mobile apps are reviewed in [5]: (1) clinical effectiveness; (2) usability and feasibility; (3) service care delivery efficiencies and economic benefits; and (4) readiness for clinic adoption.

In [6], remote assessments for early signs of autism using mobile and video applications were reviewed. The mobile apps were used to collect data using ecological moment assessments (EMAs) [7].

In [8], studies on using augmented reality technology to facilitate learning of children and adolescents with ASD were reviewed. Mobile apps with some augmented reality elements were used to help individuals learn a number of skills, such as attention management, navigation, facial expression and emotions, brushing teeth, literacy, and social communication. In [9], a review on the application of mobile augmented reality in ASD intervention was presented. Although different research questions are explored, the studies included in the the review are almost identical to those included in [8].

In [10], a systematic review focused on the self-monitoring, treatment, and data collection in children, adolescents, and young adults with psychiatric disorders using mobile apps. The review considered a large array of psychiatric disorders, such as substance addictions, depression, ADHD, etc. The studies surveyed are generally positive on the efficacy of using mobile apps for intervention.

All these reviews are centered around the efficacy of various interventions that incorporate mobile apps in some way. Previously, we reviewed the roles played by general technologies in autism care [11]. In this paper, we focus on reviewing the design and implementation of the mobile apps for autism care. For completeness, we also include some other aspects of autism care that are typically included in systematic reviews. Specifically, this review is guided by the following research questions:

RQ1 : Who are the beneficiaries of these mobile apps?
RQ2 : What are the primary purposes of these mobile apps?
RQ3 : What mechanisms are used in these mobile apps to improve user engagement?
RQ4 : What guidelines are followed in mobile app development?
RQ5 : What are the theoretical foundations in the mobile app design to ensure intervention effectiveness?

The remaining of the paper is organized as follows. Section 2 outlines the method of literature collection. Section 3 reports our findings for the first research question regarding the beneficiaries of the mobiles apps. Section 4 elaborates the primary purposes of the mobile apps, which constitutes the findings for our second research question. Section 5 summarizes the mechanisms incorporated in the mobiles app to engage users (i.e., our findings for our third research question). Section 6 documents the software development guidelines used in the design and implementation of the mobile apps (findings for the fourth research question). Section 7 provides the various theories and frameworks that guided the intervention design (findings for the fifth research question). Section 8 synthesizes our findings and expresses our observations. Section 9 concludes this paper.

## 2. Method of Literature Collection

**Figure 1:**
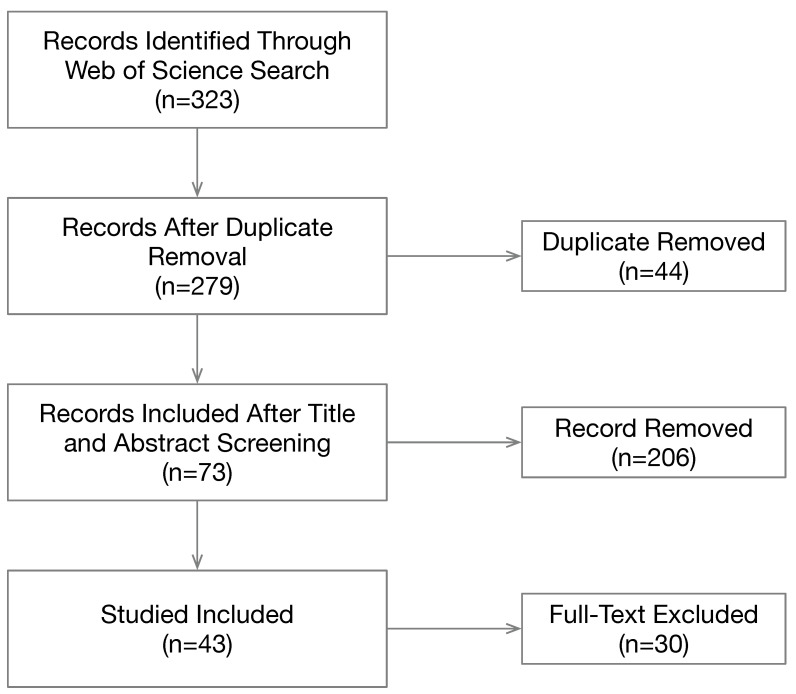
Literature selection process.

We choose to use the Web of Science core collection as the main vehicle to find relevant publications because this database is the most authoritative and comprehensive source for academic publications due to its high standard. We used four search terms: “autism mobile app”, “asd mobile app”, “autism smartphone”, and “asd smartphone”. The search returned a total of 323 publications. The selection process is illustrated in Figure 1. Then, 44 duplicate records were removed. Next, we screened the remaining records by inspecting the title and abstract of each record, which eliminated 206 records that we deemed as irrelevant. The full texts of the remaining 73 records were then retrieved for further evaluation. We removed 30 records based on the following criteria: (1) published in English; (2) at least six pages long; (3) dedicated to autism study with substantial technical details or clinical results; and (4) mobile apps play a major role in the study. This review is based on 43 publications.

Other than setting a basic set of criteria for the inclusion of publications, we intentionally did not set further metrics to rank the included publications. We also intentionally did not limit our review to formal intervention studies with controlled and randomized trials because autism is a life-long disorder and individuals with ASD often require many forms of help beyond formal clinical interventions from various caregivers (such as parents, family members, and school teachers). Furthermore, although a large number of mobile apps have been developed for individuals with ASD, research in this area is still in its infancy. Hence, we included studies that explore the software development issues with no or very limited human subject trials. Furthermore, we included four studies on assessing the quality of educational mobile apps for children with ASD.

## 3. RQ1: Who Are The Primary Beneficiaries of the Mobile Apps?

There are four types of beneficiaries of the mobile apps: individuals with ASD, parents and family members of the individuals with ASD, teachers and therapists of the individuals with ASD, and clinicians/researchers who are diagnosing and/or assessing ASD. We use the term “beneficiaries” instead of users because some mobile apps are designed to collect data regarding the behaviors and status of the individuals with ASD while they are using the apps. For these mobile apps, although the users are individuals with ASD, the primary purpose is for data collection. We also chose not to use the term “target population” because it has specific meaning in the field of healthcare and medicine and it is usually defined in formal controlled trials. Although there are four different types of beneficiaries, all the studies target the diagnosis, assessment, care, and intervention of the autism community.

**Figure 2:**
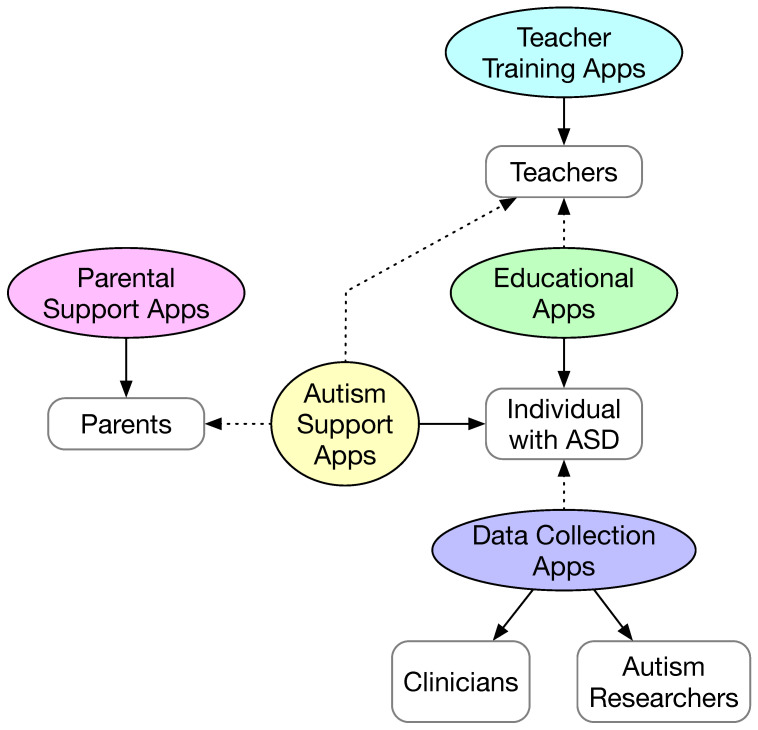
Major types of mobile apps. The beneficiaries of the apps (i.e., the primary users for all but the data collection apps) are denoted by solid arrows. Other users of the apps are denoted by dotted arrows.

The taxonomy of the mobile apps and their beneficiaries are shown in Figure 2. We categorize the mobiles apps into five types: (1) autism support apps; (2) educational apps; (3) parental support apps; (4) teacher training apps; and (5) data collection apps. The individuals with ASD are further divided into children, adolescents (i.e., young adults), college students, and adults. Individuals with ASD are the primary users and beneficiaries of autism support apps and educational apps. Parents and teachers may be the secondary users of the autism support apps to provide content and customize the apps. Educational apps may also allow the teachers to configure and customize the apps as the secondary users. Educational apps are typically used in a classroom setting under the guidance of teachers.

In Figure 2, the beneficiary of each type of app is indicated using a solid arrow. In addition to the primary users, some types of apps have secondary users, for example, to customize the app content for the primary app user. For all types of apps, except the data collection apps, the primary user is also the beneficiary. Although the individuals with ASD are the primary users of the data collection apps, the beneficiary of such apps is actually clinicians and autism researchers. Table 1 summarizes the primary user, the secondary user (if any), and the beneficiary for each type of mobile app and the corresponding references.

As can be seen in Table 1, the great majority of studies are about mobile apps designed for individuals with ASD (22 studies for autism support apps and 7 studies for education apps). However, there are studies that target other beneficiaries. Among the forty-three studies, two studies target school teachers, four studies target parents, family members, and therapists, and eight studies focused on using mobile apps to collect data for clinicians and autism researchers towards early diagnosis and assessment of autism.

## 4. RQ2: What Are the Primary Purposes of the Mobile Apps?

**Figure 3:**
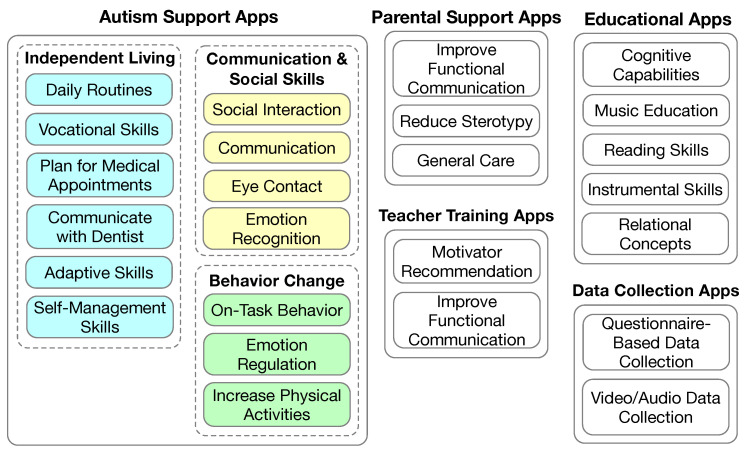
The target skills of the mobile apps.

The major types of mobile apps and their primary purposes are illustrated in Figure 3. Autism support apps are designed for individuals with ASD to learn one or more specific skills by themselves. Most educational apps are designed to facilitate the learning of curriculum subjects such as reading and math. However, educational apps may also target non-academic skills such as social communication skills. Parental support apps are designed to train parents on how to provide care for their children with ASD, and also care for themselves while providing care to their children with ASD. Teacher training apps are designed to train teachers of students with ASD. Data collection apps are designed to collect data for clinicians and autism researchers. Some data collection apps are designed to collect data while they are used by individuals with ASD. Other data collection apps are designed for the clinicians or autism researchers to record data while observing the individuals with ASD.

### 4.1. Parental Support Apps

The studies on parental support apps are summarized in Table 2. In [41], a mobile app called Smartautism was used to guide the parents to provide the necessary parental support to their children with ASD in between medical appointments. The mobile app consists of two components: (1) an ecological momentary assessment of the children with ASD and the psychological state of the parents themselves and (2) a graphical display of the scores obtained in the ecological momentary assessment as a form of feedback to the parents. Questions in the ecological momentary assessment regarding the child with ASD cover four aspects of the child’s daily life: (1) behavior; (2) meal time pleasantness; (3) sleep quality; and (4) communication, relationships, and interactions with others. The visual feedback provided to the parents could guide the parents to alter their educational approach when necessary. The study focused on the evaluation of the acceptability of the Smartautism app with 65 participants during a 6 month period. The participants were expected to answer the questions at least twice a week.

While most of the studies are about how to help parents to take care of their children with ASD, it is also important to help parents to cope with the tremendous stress while taking care of their children with ASD. A StressLess mobile app was developed and its efficacy was studied in a randomized controlled trial [44]. The design of the StressLess app was guided by the principles of cognitive behavioral training to provide psychoeducation and interactive exercises.

In [43], a mobile app called Map4speech was used in a parent training program to improve their children’s functional communication. The app used the behavior modeling training as the conceptual framework, and it consists of four parts after introduction: (1) follow the child’s lead; (2) imitate and animate while playing with the child; (3) create moments of togetherness; and (4) verbal, gestural, and physical prompts and reward and praise the child. During the training, feedback was provided to the parents via Skype sessions.

In [42], a mobile app called iSTIM (short for individualized stereotypy treatment integrated modules) was used to support the parents of children with ASD in reducing stereotypy behaviors (i.e., repetitive vocal and motor behaviors). The iSTIM app consists of four modules: (1) a set of preliminary questions regarding the child and the stereotypy behavior of the child; (2) baseline data collection on the child’s stereotypy behavior; (3) identification of preferred stimuli; and (4) definition of the intervention procedure. The study itself focused on the validation of the decision-making algorithms, which are used to recommend a preferred stimuli and intervention procedure. Instead of recruiting parents of children with ASD, student research assistants used the iSTIM with 11 children who have ASD.

### 4.2. Teacher Training Apps

The two studies on teacher training apps are summarized in Table 3. In [46], the Map4speech mobile app was used to train teachers in a very similar fashion to the authors’ earlier study [43], where the app was used to train parents to improve the functional communication of their children with ASD.

In [45], the usability and efficacy of a mobile app called IEP-Connect were reported. IEP in the app name stands for individualized education program. The study was conducted in the context of classroom education. The app was designed to select the most optimal motivator for teachers and therapists to use based on the recorded problematic behaviors of their students. The motivator selection is based on an applied behavior analysis and incorporates reinforcement learning. The decision making is based on Markov decision processes. The effectiveness of the motivator selected is measured by the student motivation after the selected motivator has been applied. The motivators consist of several categories, including editor items (e.g., fruits or snacks), sensory items (e.g., listening to music or playing with sand), activity items (e.g., drawing or playing computer games), token items (e.g., stickers or money), social items (e.g., high fives or praise), and choice (e.g., the student is given the chance to choose what to use or do). The teachers are supposed to observe student reactions once the motivator is applied to the student.

### 4.3. Autism Support Apps

A large number of mobile apps have been developed to help individuals with ASD to learn, live independently, work productively, and live a normal life with social interactions. All these apps can be regarded as autism support apps. Some of these apps are designed to be used in a classroom setting. Others are designed to be used in other settings. The former will be reviewed in the next section separately. In this section, we focus on the latter. When we say autism support apps, we also mean the latter apps. We divide these mobile apps into three categories: (1) communication and social interaction skills; (2) independent living skills; and (3) behavior changes.

#### 4.3.1. Communication and Social Interaction

The mobile apps for promoting communication and social interaction are summarized in Table 4. In [12], a mobile app called MyCalendar was used to help children with ASD who have limited verbal skills to communicate with their teachers in schools and with their families at home. The app presents a calendar view to the users, where the children, teachers, and family members may add photos and videos to the calendar each day. The mobile app encourages the children with ASD to communicate with the teachers at school and the family members at home. The app also allows the users to annotate the photos and videos, which make the app a visual diary of the children with ASD. That said, the main goal of the app is to encourage children with ASD to communicate with their teachers and family members, and reinforce the value of social interaction and emotional bonds via replaying and modeling positive social interactions.

In [13], two mobile apps were used to encourage social interactions in children with ASD. The first app helps children to learn emotions and facial expressions. The second app helps children to understand social interactions. The app design incorporated ideas from comic books, where a social interaction scenario is illustrated using a sequence of images and from an earlier computer educational game for children with autism called TouchStory [56], where events in pictures were used as discrete stimuli to help children with ASD to develop coherent narratives.

In [14], a mobile app called Ying was developed to improve the communication and social skills of children with ASD. The app contains four games for emotion recognition. A notable novelty of the study is the use of the child’s own photos in the games with automatic emotion recognition using the Microsoft Emotion cloud application programming interface (API).

In [15], a mobile app called Yuudee was developed and evaluated to facilitate minimally verbal children with ASD to make requests. The app provides augmentative and alternative communication via pictures. More specifically, the picture exchange communication system (PECS) [57] was adopted in the app as a means for augmentative and alternative communication. The PECS enables a minimally verbal child with ASD to communicate with others by exchanging pictures.

In [17], a mobile app called Sidekick! was developed and used to teach a child with ASD basic skills and facilitate communication in therapy sessions, at school, and at home. A unique feature of the app is allowing the child to choose an avatar that aligns with their restricted interest.

In [29], a messaging app (multimodal messaging, or MAAM) was developed and evaluated for adults with ASD. The app incorporates text-to-speech, speech-to-text, and communication symbols to encourage users to engage in communication and improve their social and communication skills. The evaluation confirmed that the use of app helped reduce social loneliness.

In [16], the development of a mobile serious game called SimpleTEA for non-verbal children with ASD was documented. The app adopts PECS as the method for enabling augmented and alternative communication. In addition, the app incorporates a voice assistant that can help the child read.

In [19], a mobile app called “We Are Friends” was used to increase eye contact during social interactions. The app consists of the daily routines of four activities with six social skill modules. To help children with ASD to generalize eye contact skills to daily living, the faces used in the app are gradually changed from all familiar ones to predominately unfamiliar ones. Video modeling was used as the guiding principle of the app design.

In [18], a mobile app called AutiSay was developed to help children with ASD to communicate with others. The app uses a PECS to facilitate communication via pictures, and provide pre-recorded audio scripts to enhance communication. This would also give children with ASD an opportunity to learn how to express their emotions and needs. In addition to facilitating communication, AutiSay also teaches children life skills and how to plan for activities.

#### 4.3.2. Independent Living

Several mobile apps have been developed to help individuals with ASD to achieve independent living, as outlined in Table 5. These not only include the training of children with ASD to perform daily routines such as brushing teeth and putting on clothes, but also include the training of more demanding skills such as planning for trips and medical appointments, self-management skills, vocational skills, and adaptive skills.

In [20], the design of a mobile app called AutiAct was reported. The app helps a child with ASD to learn some basic daily routines at three different difficulty levels: easy, medium, and hard. The easy level consists of the daily routines of washing their hands and face. The medium level consists of the daily routines of brushing their teeth and flushing the toilet. The difficult level consists of one daily routine of putting on shoes. For each daily routine, a training video is provided in the app. The underlying mechanisms adopted in the app are augmentative and alternative communication [58] and video modeling [59].

In [23], a controlled case study was reported using a mobile app called VideoTote for the parent of the participant (a 17-year-old female with ASD) to make video recordings of some daily routines (requesting for help in a store, checking out in a supermarket, and ordering food in a restaurant) for the participant to learn. In the videos, the parent acted as a store clerk, cashier, and restaurant employee while performing the related routines. Video modeling is used as the underlying mechanism of the study.

In [24], a mobile app running on a tablet was developed to enhance the independent living skills of adolescents with ASD in a vocational setting. The app design chooses to use video modeling enhanced with integrated visual scene displays as the intervention method. A visual scene display refers to imbedding an image that denotes some meaningful event to the trainee as a hot spot. The vocational tasks take place in a school library setting, including checking in books, putting books away, making dye-cut prints, and paper shredding. For each task, the trainee is supposed to ask the library staff for permission and inform the library staff of their intent to perform a task and the completion of a task.

In [21], a mobile app called PlanTEA was developed to help plan and anticipate medical appointments for children with ASD. The app provides two functionalities: (1) make plans on what to do before, during, and after each medical appointment using anticipation boards and (2) plan management. The design of the app followed a list of recommendations for software intended for users with ASD. The intervention methodology is based on providing pictogram-based anticipation boards, and augmentative and alternative communication.

In [30], a mobile app called Virtuoso was developed to help adults with ASD to learn adaptive skills. Adaptive skills refer to the capability of making the necessary adjustment when the individual’s environment is changed, which is essential for independent living and social interactions. The app provides an immersive, spherical, video-based virtual reality. The intervention foundation is video modeling. The use of a spherical virtual reality interface is to make the app more engaging. More specifically, the app was designed as a public transportation app with four tasks: (1) find the location of the shuttle station; (2) go to the shuttle station; (3) check the schedule of the shuttle with another app; and (4) get on the shuttle when it arrives.

In [25], a mobile app called LifePal was developed to help adolescents with ASD to perform self-management of their life, including daily task management (such as personal hygiene tasks and school tasks), daily life logs where the individual records their ongoing mood, actions, and thoughts, local commute, and emergency calling. The app encourages the involvement of parents of the adolescents, for example, to verify the completion of tasks and to set up geo-fencing so that the parents would know the whereabouts of their children. The app incorporates some gamification and positive reinforcement mechanisms to enhance its engagement level.

In [22], a mobile app was developed to facilitate communication between a child with ASD and a dentist during their first visit. The app appears to be based on a picture exchange communication system and consists of a sequence of screens specific to a dental visit.

#### 4.3.3. Behavior Changes

Several mobile apps have been developed to improve the behavior of individuals with ASD, as shown in Table 6, typically by applying well-established best practices in behavioral changes [60,61,62,63]. The target behavior changes include emotion regulation, self regulation, and being more physically active.

In [27], an online platform with both a mobile app and a web interface was used to support high school and college students with ASD in their academic studies. The goal of the study was to improve the students’ self-determination and goal setting by allowing the student to carry out self-directed study. The system consists of an interactive learning management module with a chat function that allows the student to reach out to clinicians for help when needed.

In [26], a mobile app was developed and evaluated to help adolescents with ASD to regulate their emotions in a classroom. The app adopts two strategies for emotion regulation: (1) visual support to increase emotional awareness and (2) co-regulation of emotions with stakeholders such as parents and teachers. The strategies are guided by the principles of cognitive behavioral therapies for emotion regulation [64].

In [31], a mobile game called PuzzleWalk was developed and evaluated for usability. The app was designed for promoting physical activity in adults with ASD. The mobile app development process was documented in great detail. The app design considered a number of factors that fit the needs of adults with ASD: (1) social connection; (2) predictability; (3) focused and filtered information; (4) awareness that the user’s cognitive levels may differ significantly; and (5) maintaining progress in the level of physical activity. In [32], the usability of the PuzzleWalk app was examined.

In [33], the feasibility of the PuzzleWalk app was evaluated in a randomized control trial. This study revealed that this app incorporated behavior change techniques by considering psychological determinants including autonomy, self-efficacy, and intrinsic and extrinsic motivation. In addition to an increased physical activity level, the trial found that the anxiety level was reduced as well.

In [28], a meta-analysis was conducted on studies using the I-Connect mobile app to increase on-task behavior. The I-Connect app was designed to allow an individual to perform self-monitoring towards the goal of improving positive behaviors. The app has been used in many studies in the college education setting involving students with ASD.

### 4.4. Educational Apps

The number of educational mobile apps is large, as indicated in [38,39]. However, academic publications on the design, implementation, and evaluation of educational apps are limited, which are outlined in this section and summarized in Table 7.

In [38], a set of criteria was developed to assess the mobile apps (limited to the Android platform) for teaching children and adolescents basic instrumental skills (oral language, reading, writing, and mathematics). The study found 88 apps (English and Spanish) from the Google Play Store. The criteria include three dimensions: design and form (availability of the app, ergonomics, usability, popularity, and accessibility), content (coverage of key elements in pronunciation, reading, writing, and math, guide on using the app, audio quality, and safety), and pedagogical aspects (interactivity, suitability of pace and learning, and assessment). Some of the criteria could provide guidance on the development of new mobile apps that target instrumental skills. However, the criteria appear to have ignored the specific needs from children and adolescents with ASD. In a similar study [39], the same research group evaluated 155 Android apps using the same set of criteria.

In [36], a mobile app called Leo con Lula was developed to teach children with ASD how to read using a global reading method. The authors argued that global reading fits the individuals with ASD better because it is a top-down approach. First, it connects images to words. Then, the words are decomposed into syllables and the corresponding sounds. The last step is the letters and their phonetics. The app was designed for classroom use and runs on a tablet. The app design was guided by augmentative and alternative communication where a pictogram is used pervasively. The app emphasizes the customizability of the app context so that it can be personalized for each child with ASD.

In [34], the efficacy of a commercial mobile app called DoBrain was evaluated in preschool children with and without developmental disabilities in a randomized controlled trial. ASD is considered as one of the developmental disabilities. DoBrain is a cognitive training program targeting cognitive capacity, a higher-level thinking capacity, and meta processing abilities. DoBrain consists of several games at different levels of difficulty. The 24 week study showed a number of mixed results: (1) the overall cognitive development in the experimental group was not superior to that of the control group; (2) cognitive domains related to imitation, perception, and gross motor actually declined in both the experimental and control groups; (3) ASD symptoms were significantly improved in the experimental group; (4) the language skills in the experimental group had no significant difference; and (5) behavioral problems were significantly reduced in the experimental group.

In [40], 15 mobile apps were selected by surveying professionals who work with children with ASD and parents of children with ASD. The mobile apps were evaluated mostly by their content using the criteria defined in Boehm-3 [65], which defines 50 basic relational concepts. The study further examined whether or not an app allows community-contributed content. Towards the end of the paper, the authors made a list of recommendations for app developers, which we will revisit in Section 6.

In [35], the development of a mobile app called Suoniamo for teaching piano to students with ASD is reported. The app shows a virtual piano keyboard and is intended to facilitate music education. The focus of the study is to investigate the best practices in designing user interfaces for autistic learners.

In [37], a mobile app called iCanLearn was developed as an educational tool for use between a teacher and a student, or a parent and a child. The app is a flashcard educational app where the teacher can create and edit slides and the learner may view slides. A particular novelty of the app is the functionality of connecting two devices so that a teacher may work with a learner closely. Another functionality of the app is that it can be used to illustrate social stories. Flashcards have been used widely as an instructional medium, and they are very versatile. Flashcards can be used to teach/learn academic subjects, as well as to improve social behaviors by applying applied behavior analyses. iCanLearn provides a platform for the teacher/parent to create content for the child with ASD to learn.

### 4.5. Data Collection Apps

Traditionally, diagnosis and assessment of symptom changes are largely based on retrospective reports from caregivers of individuals with ASD. Such an approach may introduce errors in reporting because of the necessity of recalling past behaviors of the individuals with ASD. It is much more desirable to collect data in real time or nearly real time. Various mobile apps have been developed for this purpose and they are summarized in Table 8.

In [47], an observational study on caregivers’ daily reporting of autism symptoms using a web and mobile app called My JAKE (short for Janssen Autism Knowledge Engine) was reported. The caregiver is expected to first complete the medical and developmental history of the child with ASD in the app. Then, on a daily basis, the caregiver is required to complete the autism behavior inventory, which consists of 73 items that cover the symptoms and behaviors of the child with ASD. The app also allows the caregiver to log additional items using the journal and event tracker. Furthermore, the app can be connected to the Microsoft health vault. The usability of the app was evaluated using a human subject trial with 144 caregivers.

In [48], the study protocol for the evaluation of a mobile app for the early detection of autism is reported. The mobile app is called ASDetect. It was developed for parents with young children to collect evidence regarding their children’s early social communication development (SACS) [53]. The app helps the parents to assess how likely it is their children (12–24 months old) suffer from autism. The app is based on the social attention and communication surveillance tool. The app contains a questionnaire with 10–15 behavioral items, which are adapted from SACS.

The focus of [49] is the classification of autism based on crowdsourced audio data using machine learning. The audio data were recorded using a mobile app called “Guess What?” while the children with ASD are playing with their parents. More specifically, the study collected 77 videos from 58 children while in gameplay using the mobile app. The videos were recorded between 2018 and 2021. Among the 58 children, 20 are children with ASD and 38 are typically developing children. Three different machine learning models (a convolutional neural network, random forest, and wav2vec 2.0) were compared. Two additional studies were conducted by the same research group [50,51] using a similar method of data collection with the same mobile app. One study [50] reported the classification of emotion based on the videos collected.

In [52], a feasibility study was reported on using an iOS mobile app called Autism & Beyond to perform automatic emotion and attention analyses. The authors argued that a major barrier to early and evidence-based diagnosis and treatment of ASD is the lack of tools that can objectively assess the behavior and emotions of young children. The mobile app consists of four brief questionnaires and four short movies as clinically informed stimuli. While watching the movies, the device’s (iPhone or iPad) camera is used to capture a video of the child’s face. Then, the landmarks of the face and the head orientations are extracted from the video using computer vision techniques. The landmarks of the face are used to determine the emotion of the child. Attention is presumably determined from the head orientation.

In [55], a mobile app called RealLife Exp was used to conduct an EMA of daily life social interactions in adolescents and young adults with ASD (and another neurodevelopmental disorder called 22q11.2 deletion syndrome). In this study, the EMA is considered as a structured diary technique to collect measures in the daily life of the participants. The study protocol had a duration of 6 days. The participant would be prompted to complete an EMA questionnaire 8 times a day semi-randomly. The same EMA questionnaire is used in the entire study. The questionnaire contains 33–38 items depending on the answers to some conditionally branched questions. These items cover a number of areas, including the affect state (i.e., emotion) and whether the respondent is alone or with someone else. If they are with some else, the level of closeness of the other people is assessed.

In [54], a mobile app called ASDTests is described. The app consists of four screening questionnaires, each is designed for a user age group: (1) infants (up to 36 months old); (2) children (4–11 years old); (3) adolescents (12–16 years old); and (4) adults (17 years old and older). Each questionnaire consists of exactly 10 questions. The data collected via the questionnaire are used to detect autism traits using logistic regression and naive Bayes algorithms. This paper also provided an overview of other mobile apps for detecting autism, including AaB, Asperger’s tests, autism and developmental disorder screening, autism tests, ASDetect [48], the Indian scale for assessment of autism, and the naturalistic observation diagnostic assessment.

Study [51] reported the identification of social engagement indicators for autism. More specifically, gaze fixation patterns and visual scanning methods were extracted from videos, and the classification was performed using a deep learning model.

**Figure 4:**
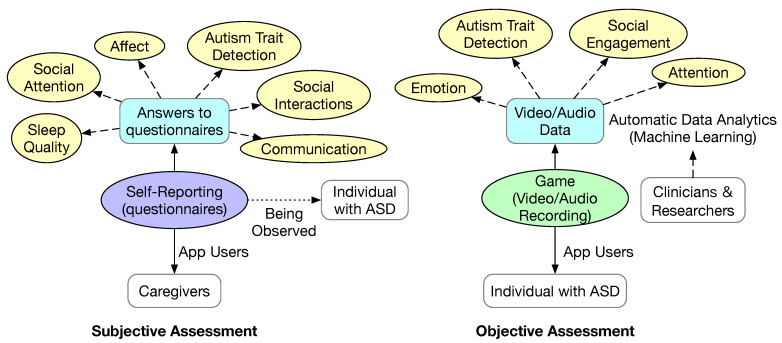
Major types of mobile apps. The primary users of the apps are denoted by solid arrows. The secondary users of the apps are denoted by dotted arrows.

From the eight studies that we included in this section, we can see that there are two major methods of data collection, as shown in Figure 4. One method is based on one or more questionnaires implemented in the mobile app. The other method is based on collecting objective data via a mobile app. The questionnaires are typically taken or adapted from standard clinical scales for autism assessment. Some questionnaire-based data collection methods follow an EMA protocol, which would prompt the user to complete a questionnaire semi-randomly throughout the day. The objective data could be audio and/or videos of the individual with ASD while playing a game as part of the mobile app. The video captured is typically of the face. It is conceivable that other data can be collected using wearable devices, such as temperature, heart rate variation, and skin resistance.

The answers to the questionnaire reflect some aspects of the daily life of the individual with ASD, such as sleep quality, affect (i.e., emotion), social interactions, social attention, and communication. The benefit of this type of mobile app is that they can facilitate the recording of these facts immediately to avoid potential mistakes in recall if the questionnaire is not completely frequently. Based on the collected answers to the questionnaire, deeper information can be extracted according to clinical scales, such as autism traits.

Based on the captured objective data, various behaviors and symptoms such as emotion, attention, and social engagement are extracted using machine learning algorithms. It is also possible to classify the likelihood of autism based on the objective data. Both traditional machine learning algorithms such as naive Bayes and deep learning have been used.

## 5. Rq3: What Mechanisms Are Used in the Mobile Apps to Improve User Engagement?

In this section, we look at non-functional mechanisms that help make the mobile apps more entertaining and engaging. The mechanisms used in the mobile apps to improve user engagement can be divided into two types: (1) mechanisms that are applicable to the general population, such as gamification and virtual reality; and (2) mechanisms that specifically fit the autism population.

### 5.1. General Purpose Mechanisms

Some mechanisms have been proven to be effective in engaging the users of mobile apps. The most prominent ones are gamification and the use of a virtual reality interface. Several mobile apps we included in this study [13,16,31,36,49,50,51] incorporated gamification. Although a single study [30] focused on the impact of using a spherical video-based virtual reality intervention to improve the adaptive skills of adults with ASD, gamification often incorporates some form of reality interfaces, such as 2D avatars [66].

#### Gamification

**Figure 5:**
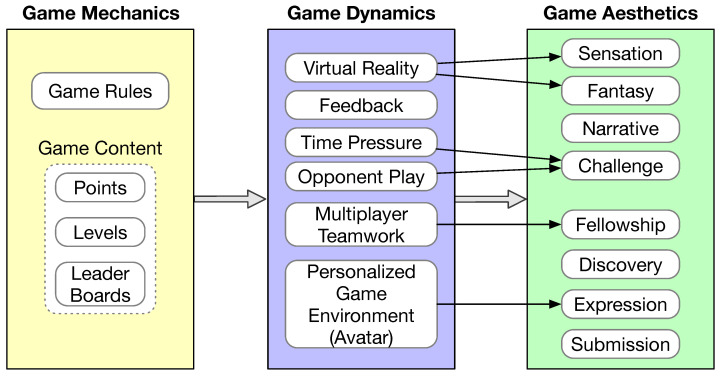
The MDA framework.

Gamification refers to adding game design elements in the mobile app. Games by definition are voluntary and enjoyable. Games are a special form of play in that games are intrinsically goal-oriented and they are bound by predefined rules [67]. Another element of games is feedback. Feedback informs the player whether or not the rules are followed and whether or not the goal has been reached. The mechanics, dynamics, and aesthetics (MDA) framework has been widely adopted in game development [68]. According to the MDA framework, game design is experience driven instead of feature driven. The game itself resembles more of an artifact instead of media. The content of the game is its behavior exhibited via the interaction with a player. The relationship between game mechanics, game dynamics, and game aesthetics is illustrated in Figure 5.

Aesthetics captures the user expectations of the game. In [68], eight aesthetics properties are defined, including sensation, fantasy, narrative, challenge, fellowship, discovery, expression, and submission. A game does not need to satisfy all eight aesthetics properties. Game mechanics are mostly defined by the rules of the game, which are inevitably game specific. Some common game content may also be considered as part of game mechanics, such as points, levels, and leader boards [67]. Game dynamics refers to how the game is implemented in a system that would give the player aesthetic experiences. Although game dynamics are game specific, there are some common elements that help achieve game aesthetics, as shown in Figure 5. Feedback is an essential component of game dynamics. Games typically incorporate some form of virtual reality, which helps achieve user sensation and fantasy. Incorporating time pressure during game play and having an opponent would increase the challenge of the game. Supporting multiple players to cooperate during game play would create fellowship experience. Allowing customization of the game environment, such as allowing the user to select an avatar or even create a unique avatar, would help satisfy the players’ expression desire.

In [69], the authors argued that game design must consider human motivations, which is particularly important for serious games developed for interventions. For example, offering rewards in the game could lead the learner to purely pursue extrinsic incentives instead of boosting intrinsic motivation and focusing on learning. Specifically, game design must be conducive to increasing relatedness, competence, and autonomy, which are the three key factors that would influence one’s intrinsic motivation as defined in the self-determination theory [70]. Ideally, the game difficulty should be kept within the range of achieving flow [71] by providing proper scaffolding when the player is experiencing difficulty to prevent frustration and anxiety and by increasing the game play difficulty level when necessary to prevent boredom.

### 5.2. Virtual Reality

Virtual reality refers to the immersive and interactive environment of a synthetic world [72]. The synthetic world might incorporate elements of the real environment to different extents. In [72], this is referred to as a virtuality continuum. For example, the virtual reality environment may use smartphone cameras and sensors to incorporate the user’s face or movement into the virtual reality scene. That is why some publications use the term “augmented” reality.

Traditionally, virtual reality is achieved via expensive 3D immersive systems such as CAVE and head-mounted goggles. Recently, devices have been made to use a smartphone in a way similar to head-mounted goggles. The Virtuoso mobile app is used in this way [30], and it was used to train adults with ASD adaptive skills. However, a virtual reality user interface does not have to be fully immersive to be effective, which has been confirmed in many autism studies [11].

### 5.3. Autism-Specific Mechanisms

Several studies incorporated autism-specific mechanisms to improve the engagement level of the mobile apps. In [17], the app design allows the user to choose an avatar that matches their own restricted interest. As a result of the alignment with the child’s restricted interest, the mobile app (called Sidekick!) was able to maintain the motivation of the child with ASD and improve the treatment adherence in therapy sessions, schools, and at home.

In [35], a mobile app is used to create a conducive learning environment for the autism community. First, for individuals with verbal communication difficulties, augmented and alternative communication should be adopted. Second, the learning environment should be error free. This is because an error would disrupt the learning process and it would require a tremendous effort of the individual with ASD to overcome the error and continue the learning process. Third, the learning environment should provide structured and facilitated teaching, i.e., the environment should provide the necessary scaffolding and reinforce positive behaviors to boost the confidence of the learner. This study advocates an accessible and minimalist design, including no background and distractors, using soft colors and fonts that have good contrast a workflow with a clear set of tasks.

In [40], the authors provided some insight into how to make mobile apps more helpful to children with ASD in the context of teaching academic skills. First, the stimuli must be provided in a clear and concrete manner. This is because children with ASD tend to pay great attention to subtle but less important details in images [73]. Second, the stimuli must be made consistent across different learning modules in color, shape, size, and reference points, again due to the tendency of children with ASD paying too much attention to unimportant details instead of the overall task.

## 6. Rq4: What Guidelines Are Followed in the Mobile App Development?

Three studies provided detailed processes in the design and development of the mobile app [16,21,31]. All of them followed a user-centered design (UCD) process [74]. UCD refers to a loosely defined process that has the following elements: (1) understand the users; (2) actively involve the users when defining the requirements; and (3) iterative design and evaluation.

To understand the users and to discover the requirements, various instruments have been used, including questionnaires, interviews, focus groups, and observations. Ideally, the targeted individuals with ASD should be the subjects of these instruments. However, it might not be practical to directly involve children with ASD in the design process due to their disorders. Some of them might be non-verbal. As such, studies often involve parents, teachers, and therapists of the children with ASD. Experienced software developers for this type of app have also been involved [16,21].

**Figure 6:**
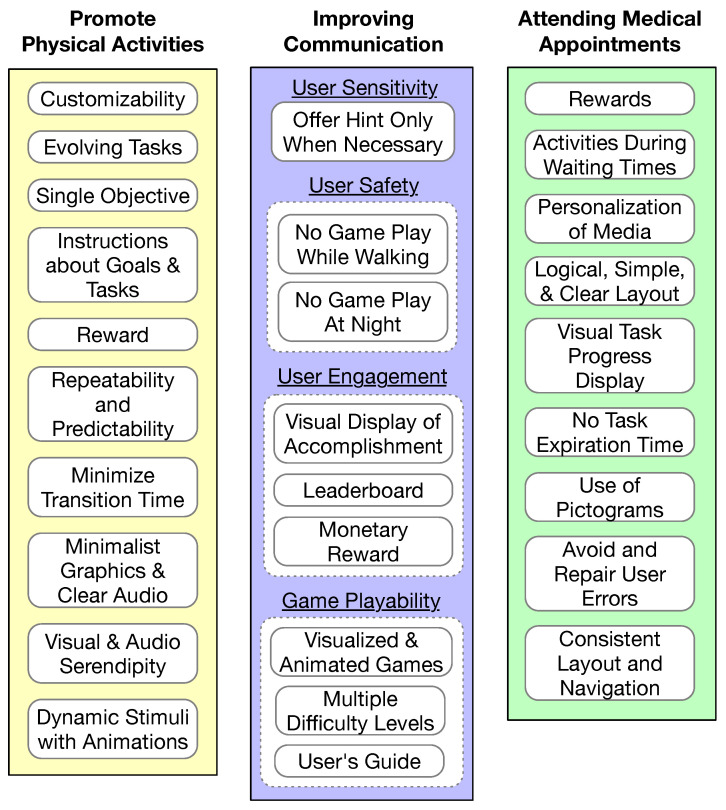
Specific design considerations for the autism population in three studies.

Each of the three studies developed or followed a set of guidelines to make the app a good fit for the target autism population and for the specific application objective, as illustrated in Figure 6. The development of the PuzzleWalk app [31], which is an app promoting physical activities for adults with ASD, included ten guidelines as shown in the left column of Figure 6. The development of the SimpleTEA app [16], which is a serious mobile game for improving communication in children with ASD, considered four major perspectives: user sensitivity, user safety, user engagement, and game playability, as shown in the middle column of Figure 6. The development of the PlanTEA app [21], which is an app supporting children with ASD to attend medical appointments, was guided by the AutismGuideline proposed in [75], as illustrated in the right column of Figure 6.

## 7. Rq5: What Are the Theoretical Foundations in the Mobile App Design to Ensure Intervention Effectiveness?

Various theories and frameworks have been used as the foundation for intervention design in mobile apps. A fraction of children with ASD are non-verbal or minimally verbal; thus, augmentative and alternative communication (AAC) [76] has been adopted in mobile apps that target these children. Even for individuals with ASD who are capable of engaging in verbal conversations, AAC may still be preferred due to their tendency of social aversion. Video modeling is also pervasively used in many mobile apps to teach individuals with ASD various skills. Mobile apps that aim to change behavior often adopt well-known behavior change techniques, cognitive behavioral training, or behavior modeling training.

### 7.1. Augmentative and Alternative Communication

**Figure 7:**
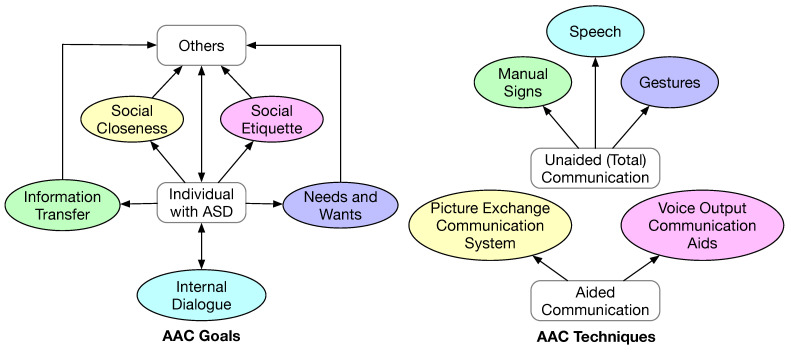
AAC goals in social interaction (**left**) and AAC techniques (**right**).

Due to the tendency of social aversion and difficulty in communication in individuals with ASD, AAC has been pervasively used in autism intervention programs. In the context of this paper, AAC refers to the methodology of helping individuals with ASD to overcome the difficulty in communication. AAC helps achieve five goals of social interaction [76], as shown in Figure 7:

- Communication of needs and wants. This is the most basic form of communication. It often involves the individual making a request to the caregiver and expecting an action-oriented response.
- Information transfer. This form of communication entails the formulation of a coherent message. Hence, it is more complex and demanding for the individual. The ability of making this form of communication is essential for an individual to receive education, stay employed, and receive healthcare.
- Social closeness. To establish friendship and other inter-personal relationships, sustained conversational communication is required. This is an even more demanding form of communication. In addition to coherent information, this form of communication requires the individual to express one’s emotions and to have a correct understanding of the feelings of the conversation partner.
- Social etiquette. This form of communication is necessary for an individual to conform to social conventions of politeness, for example, the individual is supposed to express appreciation when being helped.
- Internal dialogue. This form of communication is to communicate with oneself, which is necessary to accept oneself, to be productive daily, to perform self-reflection, and to improve oneself.

AAC techniques can be divided into unaided and aided communication [77], as shown in Figure 7. Unaided communication is also referred to as total communication. In total communication, the individual would augment speech (if any) with manual signs and gestures. In aided communication, the individual would use physical objects, pictures, drawings, or technology to augment speech or as an alternative to speech. A PECS is one of the most popular aided communication techniques. Many mobile apps implement a digital version of a PECS to aid communication. Voice output communication aids (VOCAs) refer to portable electronic devices that generate synthetic digital speech, which is another form of popular aided communication techniques. With the development of the text-to-speech technology, a mobile app could easily perform the VOCA functionality. The studies that have incorporated some form of AAC technique are summarized in Table 9.

### 7.2. Video Modeling

Video modeling is considered a form of evidence-based practice in autism intervention [78]. Video modeling refers to using video clips to demonstrate how to complete a task or the desirable behavior in a social interaction. Video self-modeling has been used in many studies, where the learner could be video-recorded while performing a task and simulating a scenario [78]. Typically, a therapist would view the recording and provide feedback. Smartphones and tablets constitute an excellent platform for an individual to acquire various skills and to gain knowledge on proper ways of social interaction via video modeling and video self-modeling. The concept of learning via modeling is attributed to Bandura, who elaborated the role of modeling in social learning [79]. As a method for autism intervention, video modeling has been used to improve social communication skills, functional skills (for daily living), and behavioral functioning [78]. In this study, video modeling has been used for children with ASD to learn basic daily routines, learn independent living skills useful in supermarkets and restaurants, learn to work in a library, take public transportation, and maintain eye contact during social interactions, as summarized in Table 10.

### 7.3. Behavior Changes

There are various behavior and behavior change theories and practices [80]. The design of the mobile apps has followed different theories and practices, including behavior change techniques [60], cognitive behavioral training [62], and behavior modeling training [63].

#### 7.3.1. Behavior Change Techniques

**Figure 8:**
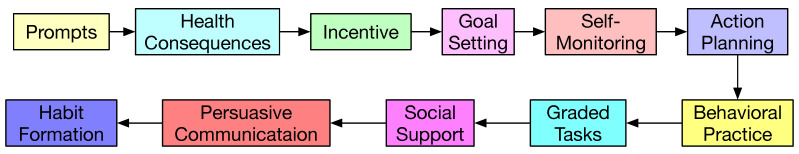
Main steps in behavior change techniques.

A large number of behavior change techniques (BCTs) have been developed [80]. A BCT includes one or more mechanisms that induce behavior changes, and typically follows a number of components as illustrated in Figure 8 [60]. The key steps of a BCT include goal setting, action planning, behavioral practice, and ultimately habit formation. The PuzzleWalk design was guided by a BCT [31,33] to promote a more physically active life style.

#### 7.3.2. Cognitive Behavioral Training

**Figure 9:**
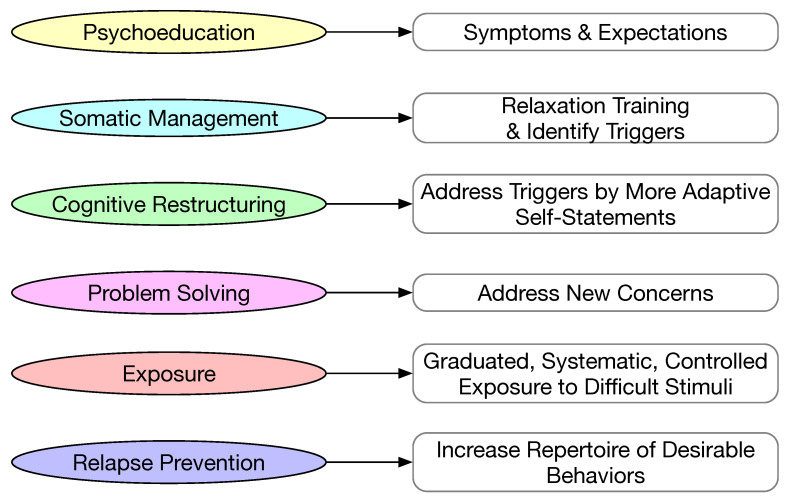
Main components in cognitive behavioral training.

Cognitive behavioral therapy (CBT) is well established in addressing psychological issues. CBT has been successfully used to reduce anxiety and improve social skills in children with ASD [62]. CBT aims to identify the cognitive conditions that result in undesirable behaviors and make corresponding improvements at the cognitive level. Generally, CBT consists of six components, as illustrated in Figure 9. The first step in CBT is psychoeducation, where the patient is educated about the symptoms of the disorder and the expectation of future conditions. Somatic management usually consists of two parts: (1) learn to relax from anxiety; and (2) identify triggers for undesirable behaviors. Cognitive restructuring is a process to transform the problematic cognitions and learn to be more adaptive when challenging scenarios occur. Problem solving is to train the patient to gain the capability of addressing unforeseen problems. Exposure is subjecting the patient to difficult scenarios in a gradual, systematic, and controlled manner so that the patient can develop coping mechanisms. Relapse prevention is to build up methods for coping with difficult stimuli with the proper response.

In [26], the design of an emotion regulation mobile app was guided by CBT. The authors adapted the original CBT for emotion regulation by following the principle that emotion regulation would require both the individual’s own desire and ability to regulate emotion and the caregiver’s help to co-regulate the emotion [64]. As such, the app incorporated two components. One component is to promote self-regulation, and the other is to facilitate emotion co-regulation. The mobile app incorporated visual elements, such as thermometer-like emotion scales and media collections for different emotion intensity levels.

In [44], the design of the StressLess mobile app was guided by CBT. The app was used in a five-week intervention to help parents of children ASD to cope with stress. The intervention consists of five components: (1) psychoeducation about stress reduction; (2) goal setting; (3) mindfulness skills; (4) positive psychology techniques and cognitive restructuring for wellbeing; and (5) positive attitude towards valued activities.

#### 7.3.3. Behavior Modeling Training

**Figure 10:**
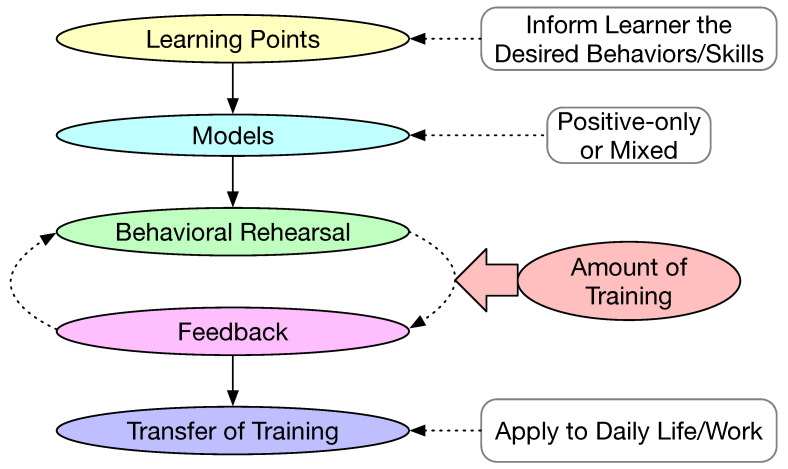
Main steps in behavior modeling training.

Behavior modeling training (BMT) is another framework for behavioral changes [63,81]. The most distinctive characteristic of BMT is modeling, which is derived from social learning theory [79]. Both positive-only models and mixed modes have been proposed and evaluated for behavioral changes [63]. The main steps in BMT are illustrated in Figure 10. The first step is to set up learning points, which is to inform the learner of the outcome of the training, i.e., the particular desired behavior or a skill to be acquired. The second step is to define the model for training. The model could be positive only or mixed. A positive-only model demonstrates the desirable behavior. A mixed model includes both desirable and undesirable behaviors. Once the models are defined, the training would proceed to asking the learner to rehearse and the trainer would provide the necessary feedback. The behavior rehearsal and feedback cycle could be iterated many times. The amount of training should also be defined as part of the intervention. The last step is to facilitate the learner to transfer the learned desired behavior to daily life or to the workplace. BMT was used as the guiding principle for the Map4speech mobile app designed to train the parents and teachers of children with ASD [43,46].

## 8. Discussion

In the previous five sections, we examined mobile apps from different perspectives. It is conceivable that individuals with ASD and people who care about them (i.e., parents, teachers, therapists, clinicians, and researchers) would want to know what apps could be helpful to address their needs. Hence, we provide a matrix of the mobile apps based on the most significant characteristics in Table 11. The matrix can help users find the particular app or set of apps that meet their needs and preferences. Data collection apps are excluded from the matrix. We also excluded several apps for teacher training and parental support that do not contain any of the characteristics.

As can be seen, the mobiles apps that we included in the current review are predominately for autism care and intervention. Only some mobile apps are used to collect data towards a diagnosis of autism. This is perhaps expected because autism symptoms vary widely from person to person. It is extremely challenging for a mobile app to collect sufficient data and make an automated diagnosis with high reliability. We still depend on experienced clinicians to make a diagnosis in the current state of the art.

A lesson learned in this review is that it takes a rigorous design process with the participation of a multidisciplinary team to develop high quality mobile apps for autism care, assessment, and intervention. The development of three apps, namely PlanTEA [21], PuzzleWalk [31], and SimpleTEA [16], indeed followed this approach. These apps were developed using a user-centered design process with a multidisciplinary team consisting of individuals with ASD and their parents, autism clinicians, autism researchers, and experienced software developers. However, most of the mobile apps were not developed in this way. Nevertheless, it is enlightening that ASD-specific software design guidelines have been proposed [75] and followed [21]. If such efforts can be standardized, the development of ASD-related mobile apps could be formalized.

We also noticed that a significant fraction of studies only included usability and feasibility validations. While such studies are informative to some extent, it is more important to clearly define ASD-specific outcomes and include data-driven validation results.

Last, but not least, mobile apps as a form of assistive technology share many common objectives and theoretical foundations in the broader scope of disability studies. For example, in [82], the authors proposed a frame that emphasizes the inter-dependency of a disabled individual with other people, which could very well be applied to the development of mobile apps for autism care.

## 9. Conclusions

In this article, we presented a systematic review on autism care, diagnosis, and intervention based on mobile apps running on smartphones and tablets. In contrast to other reviews on similar topics, which have largely focused on the clinical efficacy of using mobile apps to deliver interventions, this review aims to examine the design and implementation of the mobile apps for autism care and intervention. More specifically, this review is guided by five research questions: (1) Who benefits the most from these mobile apps? (2) What are the primary purposes of these mobile apps? (3) What mechanisms have been incorporated in these mobiles apps to improve usability? (4) What guidelines have been used in the design and implementation of these mobile apps? (5) What theories and frameworks have been used as the foundation for these mobile apps to ensure the intervention effectiveness.

Guided by the research questions 1 and 2, we proposed a taxonomy for the mobile apps used for autism care and intervention. According to our taxonomy, there are five types of mobiles apps and four types of app users. The great majority of mobiles apps are developed to help individuals with ASD. Those that are predominately used in the classroom are referred to as educational apps, and those that are primarily used at home by the individuals with ASD are referred to as autism support apps. Both types of apps may also allow the parents and teachers to access them as secondary users. There are also apps developed to train teachers to more effectively educate children and adolescents with ASD, which we refer to as teacher training apps. Similarly, parental support apps have been developed to help parents provide proper care to their children with ASD. One app was developed to reduce the stress level of parents while taking care of children with ASD. We put it in the category of parental support apps because parents will not be able to provide proper care if they themselves are stressed out. Another category is data collection apps, which are used to collect self-reported data about individuals with ASD in real time and to collect objective data about individuals with ASD. Although the primary users of some data collection apps are individuals with ASD, the beneficiaries are clinicians and autism researchers. Most apps are developed to support children with ASD, while there are also apps to help adolescents, college students, and adults with ASD.

We documented the mechanisms used in mobile apps to improve user engagement as the findings for the third research question. Gamification and virtual reality are two general purpose mechanisms for improving user engagement. Game mechanics and game dynamics together could accomplish a number of game aesthetics, such as sensation, fantasy, narrative, and challenge, which are the root reason for a higher level of user engagement. Virtual reality is usually used to provide an immersive user interface, and it is often used in conjunction with gamification. Virtual reality could be particularly attractive to individuals with ASD. In addition to gamification and virtual reality, a number of autism-specific mechanisms have been incorporated in some mobile apps, which is important because most mobiles apps were developed for individuals with ASD. These mechanisms accommodate the special needs of individuals with ASD, such as restrictive interests and the intolerance of errors.

The findings for the fourth research question are about the software development guidelines used in the design and implementation of mobiles apps. These guidelines are closely related to the mechanisms used to improve user engagement. User-centered design is the most popular process for the development of mobile apps. The intended users are involved in all stages of the development. Initially, users are consulted in the form of questionnaires, interviews, and focus groups so that the developers of the mobile apps have a good understanding of the users and what they need. Later on during the development process, users are also consulted to obtain their feedback on the prototypes. Depending on the specific aim of the app, additional considerations are incorporated in the development, some of which overlap with the autism-specific mechanisms for improving user engagement.

Guided by the fifth research question, we compiled the theories and frameworks used as the foundation for the intervention design in the mobile apps. To facilitate communication, augmentative and alternative communication is the de facto standard in mobile app design. For skills learning, video modeling has been pervasively used. For behavior change mobile apps, various techniques and methods have been adopted as the foundation for intervention design. Perhaps due to the complexity of behavior changes, there is no single universally accepted strategy. Many different theories and techniques have been proposed, and each has its followers.

## Figures and Tables

**Table 1:** Main types of the mobile apps and different types of users.

App Type	Beneficiary	Primary User	Secondary User	References
Autism Support (n = 22)	Children with ASD	Children with ASD	Parents/Teachers	[12,13,14,15,16,17,18,19,20,21,22]
	Adolescents with ASD	Adolescents with ASD	Parents/Teachers	[23,24,25,26,27]
	College Students with ASD	College Students with ASD	N.A.	[27,28]
	Adults with ASD	Adults with ASD	N.A.	[29,30,31,32,33]
Educational (n = 7)	Children with ASD	Children with ASD	Teachers	[34,35,36,37,38,39,40]
Parental Support (n = 4)	Parents	Parents	N.A.	[41,42,43,44]
Teacher Training (n = 2)	Teachers	Teachers	N.A.	[45,46]
Data Collection (n = 8)	Clinicians and Autism Researchers	Children with ASD	Clinicians and Autism Researchers	[47,48,49,50,51,52,53,54]
	Clinicians and Autism Researchers	Adolescents with ASD	Clinicians and Autism Researchers	[55]

**Table 2:** Studies on parental support apps.

App Name	Beneficiaries	Study Aim	References
Smartautism	Parents of children with ASD	General parental support	Bonnet et al., 2021 [41]
StressLess	Parents of children with ASD	Help parents of children with ASD to reduce stress	Fuller-Tyszkiewicz et al., 2020 [44]
Map4speech	Parents of children with ASD	Guide parents to improve their children’s functional communication	Law et al., 2018 [43]
iSTIM	Parents of children with ASD	Guide parents to an appropriate intervention procedure in reducing children’s stereotypy behavior	Préfontaine et al., 2019 [42]

**Table 3:** Studies on teacher training apps.

App Name	Beneficiaries	Study Aim	References
Map4speech	Teachers of students with ASD	Guide teachers to improve their students’ functional communication	Law et al., 2019 [46]
IEP-Connect	Teachers and therapists of children with ASD	Motivator recommendation	Siyam et al., 2022 [45]

**Table 4:** Mobile apps for communication and social interaction.

App Name	Target Skills	Mechanisms	References
MyCalendar	Communication with teachers and family members	Reinforce the value of social interaction with annotated photo and video	Abdullah et al., 2016 [12]
N. A.	Social interaction skills	Comic books and TouchStory	Ahmad et al., 2015 [13]
Ying	Facial and emotion recognition	Use of user’s own photos and automatic emotion recognition	Alharbi et al., 2020 [14]
Yuudee	Making requests	Picture exchange communication system	An et al., 2017 [15]
Sidekick!	General skills and communication	Avatar that aligns with the child’s restrictive interest (i.e., trains)	Birtwell et al., 2019 [17]
MAAN	Communication	Text-to-speech, speech-to-text, and communication symbols	Hijab et al., 2021 [29]
SimpleTEA	Communication	Picture exchange communication system; gamification	Jaramillo-Alcázar, et al., 2022 [16]
We Are Friends	Eye contact skills	Video modeling	Polcycrhonis et al., 2022 [19]
AutiSay	Communication and life skills	Picture exchange communication system	Voon et al., 2015 [18]

**Table 5:** Mobile apps for independent living.

App Name	Target Skills	Mechanisms	References
AutiAct	Perform daily routines	Augmentative and alternative communication and video modeling	Ahmad et al., 2020 [20]
VideoTote	Independent living	Video modeling	Allen et al., 2015 [23]
Easy VSD	Independent participation in vocational activities	Video modeling with visual scene displays	Babb et al., 2019 [24]
PlanTEA	Plan and anticipate medical appointments	Pictogram-based anticipation boards; augmentative and alternative communication	Hernandez et al., 2022 [21]
Virtuoso	Adaptive skills	Spherical virtual reality and video modeling	Schmidt et al., 2021 [30]
LifePal	Self-management skills	Gamification and positive reinforcement	Skilllen et al., 2016 [25]
N.A.	Facilitate communication between child and dentist	Augmented and alternative communication	Zink et al., 2018 [22]

**Table 6:** Mobile apps for behavior changes.

App Name	Target Skills	Mechanisms	References
N.A.	Improve self-determination and goal setting	Self-directed interactive learning platform with clinical support	Bellon et al., 2021 [27]
ER app	Emotion regulation	Emotion identification and co-regulation with stakeholders	Fage et al., 2019 [26]
PuzzleWalk	Increase physical activity	Gamification and behavior change techniques	Kim et al., 2020 [31]; Lee et al., 2020 [32]; Lee et al., 2022 [33]
I-Connect	On-task behavior	Conduct self-monitoring	Scheibel et al., 2022 [28]

**Table 7:** Educational mobile apps.

App Name	Target Skills	Notes	References
88 apps and 155 apps	Basic instrumental skills (oral language, reading, writing, math)	Assessing the apps in the Play App Store	Gallardo et al., 2021 [38]; Gallardo et al., 2022 [39]
Leo con Lula	Reading skills	Use of global reading method; based on augmentative and alternative communication	Gomez et al., 2018 [36]
DoBrain	Cognitive capabilities	Randomized controlled trail with mixed results	Lee et al., 2022 [34]
15 apps	Basic relational concepts	Assessing the apps based on the coverage of basic relationship concepts	Mykyta et al., 2017 [40]
Suoniamo	Music education	Focused on the design of user interfaces for the autism learners	Senette et al., 2021 [35]
iCanLearn	General academic knowledge	Flashcard application; may connect devices used by teacher and learner	Zaffke et al., 2015 [37]

**Table 8:** Mobile apps for data collection.

App Name	Beneficiaries	Study Aim	References
My JAKE	Clinicians (caregivers are the users of the app)	Record the sleep quality, affect, and other specific behaviors of a child with ASD via a questionnaire	Bangerter et al., 2019 [47]
ASDetect	Parents of children with ASD and clinicians	Record social attention and communication behaviors of children for detection of autism via a questionnaire	Barbaro et al., 2020 [48]
Autism & Beyond	Clinicians and researchers	Automatic emotion and attention analysis based on videos captured while children with ASD are watching clinically informed short videos	Egger et al., 2018 [52]
RealLife Exp	Clinicians and researchers	Collecting data regarding daily life social interactions using EMA	Feller et al., 2023 [55]
ASDTests	Clinicians and parents	Autism screening using a 10 question questionnaire with automatic detection of autism traits.	Thabtah, 2019 [54]
Guess What	Researchers (app was used to record audio data of the children with ASD while they are playing the game)	Autism assessment based on the audio data collected via the app	Chi et al., 2022 [49]
	Same as above	Emotion classification based on the video data collected via the app	Kalantarian et al., 2020 [50]
	Same as above	Identification of social engagement indicators for autism based on the videos collected via the app	Varma et al., 2022 [51]

**Table 9:** AAC usage in mobile apps.

AAC Technique	Intervention Goals	App Name	References
PECS (photos and videos)	Social closeness (social interaction with family and teachers)	MyCalendar	Abdullah et al., 2016 [12]
PECS (comic books and touchstory)	Prerequisite for social closeness (facial emotion recognition)	Ying	Alharbi et al., 2020 [14]
PECS	Need and wants	Yuudee	An et al., 2017 [15]
PECS (pictograms)	General communication	Sidekick!	Birtwell et al., 2019 [17]
PECS (pictograms)	Needs and wants, information transfer	PlanTEA	Hernandez et al., 2022 [21]
PECS (Communication symbols) and VOCA (text-to-speech and speech-to-text)	General communication	MAAN	Hijab et al., 2021 [29]
PECS	General communication	SimpleTEA	Jaramillo-Alcázar et al., 2022 [16]
PECS (pictograms)	General communication	AutiSay	Voon et al., 2015 [18]
PECS (flashcard) and VOCA (audio recordings)	Needs and wants, information transfer	N.A.	Zink et al., 2018 [22]

**Table 10:** Video modeling usage in mobile apps.

Target Skills	Video Production	App Name	References
Daily routines (washing hands and face; brushing teeth and flushing the toilet; putting on shoes)	Presumably the researchers made the videos for modeling; the app allows the learners to perform self-modeling	AutiAct	Ahmad et al., 2020 [20]
Independent living (requesting help in a store, checking out in a supermarket, and ordering food in a restaurant)	Parent and participant recorded videos together in a mock setup	VideoTote	Allen et al., 2015 [23]
Vocational skills (working in a library)	Researchers made the videos; the videos are enhanced with visual scene displays	Easy VSD	Babb et al., 2019 [24]
Eye contact during social interaction	Recorded by the researchers while simulating specific social scenarios	We Are Friends	Polcycrhonis et al., 2022 [19]
Using public transportation	Presumably the researchers made the videos for training	Virtuoso	Schmidt et al., 2021 [30]

**Table 11:** Mobile app feature matrix.

AppName		Purpose						Engagement			Theoretical Foundation				
		CS	IL	BC	Edu	PS	TT	VR	Gm	As	AAC	VM	BCT	CBT	BMT
MyCalendar		✓									✓	✓			
Ahmad15 *		✓							✓		✓				
Ying		✓							✓		✓				
Yuudee		✓									✓				
MAAN		✓									✓				
SimpleTEA		✓							✓		✓				
Sidekick!		✓								✓					
AutiSay		✓									✓				
We Are Friends		✓										✓			
AutiAct			✓								✓	✓			
VideoTote			✓									✓			
Easy VSD			✓									✓			
PlanTEA			✓								✓				
Zink18 *			✓								✓				
Virtuoso			✓					✓			✓				
LifePal			✓						✓						
I-Connect				✓											
ER app				✓										✓	
PuzzleWalk				✓					✓				✓		
Bellon21 *				✓								✓			
DoBrain					✓				✓						
Suoniamo					✓					✓	✓				
Leo con Lula					✓					✓	✓				
iCanLearn					✓						✓				
Map4speech						✓	✓				✓				✓
StressLess						✓					✓			✓	

CS: communication and social skills; IL: independent learning skills; BC: behavior changes; Edu: educational apps; PS: parental support; TT: teacher training; VR: virtual reality; Gm: gamification; As: ASD-specific mechanisms; AAC: augmentative and alternative communication; VM: video modeling; BCT: behavior change techniques; CBT: cognitive behavioral training; BMT: behavior modeling training. The checkmark ✓ in the matrix means that the mobile app has the particular feature. * Note: for apps that do not have a name, the last name of the first author and the two digit publication year are used together as the app names.

## Data Availability

Not applicable.

## Abbreviations

The following abbreviations are used in this manuscript:

ASD	Autism Spectrum Disorders
AAC	Augmentative and Alternative Communication
ADHD	Attention Deficit/Hyperactivity Disorders
BCT	Behavior Change Techniques
BMT	Behavior Modeling Training
CBT	Cognitive Behavioral Therapy
EMA	Ecological Momentary Assessment
MDA	Mechanics, Dynamics, and Aesthetics Framework
PECS	Picture Exchange Communication System
SACS	Social Communication Development
UCD	User-Centered Design
VOCA	Voice Output Communication Aid

## References

[1] Silva B.M., Rodrigues J.J., de la Torre Díez I., López-Coronado M., Saleem K. (2015). Mobile-health: A review of current state in 2015. J. Biomed. Inform..

[2] Zhao W., Luo X., Qiu T. (2017). Smart healthcare. Appl. Sci..

[3] Lord C., Cook E.H., Leventhal B.L., Amaral D.G. (2000). Autism spectrum disorders. Neuron.

[4] Kanner L. (1943). Autistic disturbances of affective contact. Nerv. Child.

[5] Valentine A.Z., Brown B.J., Groom M.J., Young E., Hollis C., Hall C.L. (2020). A systematic review evaluating the implementation of technologies to assess, monitor and treat neurodevelopmental disorders: A map of the current evidence. Clin. Psychol. Rev..

[6] Dahiya A.V., McDonnell C., DeLucia E., Scarpa A. (2020). A systematic review of remote telehealth assessments for early signs of autism spectrum disorder: Video and mobile applications. Pract. Innov..

[7] Shiffman S., Stone A.A., Hufford M.R. (2008). Ecological momentary assessment. Annu. Rev. Clin. Psychol..

[8] Khowaja K., Banire B., Al-Thani D., Sqalli M.T., Aqle A., Shah A., Salim S.S. (2020). Augmented reality for learning of children and adolescents with autism spectrum disorder (ASD): A systematic review. IEEE Access.

[9] Lian X., Sunar M.S. (2021). Mobile augmented reality technologies for autism spectrum disorder interventions: A systematic literature review. Appl. Sci..

[10] Melbye S., Kessing L.V., Bardram J.E., Faurholt-Jepsen M. (2020). Smartphone-based self-monitoring, treatment, and automatically generated data in children, adolescents, and young adults with psychiatric disorders: Systematic review. JMIR Ment. Health.

[11] Liu X., Wu Q., Zhao W., Luo X. (2017). Technology-facilitated diagnosis and treatment of individuals with autism spectrum disorder: An engineering perspective. Appl. Sci..

[12] Abdullah M.H.L., Wilson C., Brereton M. Mycalendar: Supporting families to communicate with their child on the autism spectrum. Proceedings of the 28th Australian Conference on Computer-Human Interaction.

[13] Ahmad M.I., Shahid S. (2015). Design and evaluation of mobile learning applications for autistic children in Pakistan. Human–Computer Interaction–INTERACT 2015: 15th IFIP TC 13 International Conference, Bamberg, Germany, September 14–18, 2015, Proceedings, Part I 15.

[14] Alharbi M., Huang S. An Augmentative System with Facial and Emotion Recognition for Improving Social Skills of Children with Autism Spectrum Disorders. Proceedings of the 2020 IEEE International Systems Conference (SysCon).

[15] An S., Feng X., Dai Y., Bo H., Wang X., Li M., Woo J.Z., Liang X., Guo C., Liu C.X. (2017). Development and evaluation of a speech-generating AAC mobile app for minimally verbal children with autism spectrum disorder in Mainland China. Mol. Autism.

[16] Jaramillo-Alcázar A., Arias J., Albornoz I., Alvarado A., Luján-Mora S. (2022). Method for the Development of Accessible Mobile Serious Games for Children with Autism Spectrum Disorder. Int. J. Environ. Res. Public Health.

[17] Birtwell K.B., Platner A.K., Nowinski L.A. (2019). Exploring the use of Sidekicks! For children with autism spectrum disorder (ASD). Psychol. Serv..

[18] Voon N.H., Bazilah S.N., Maidin A., Jumaat H., Ahmad M.Z. (2015). Autisay: A mobile communication tool for autistic individuals. Computational Intelligence in Information Systems: Proceedings of the Fourth INNS Symposia Series on Computational Intelligence in Information Systems (INNS-CIIS 2014).

[19] Polychronis S.C., Johnson A., Thelin R.J., Eggett D.L., Christensen J. (2022). Use of an App With Embedded Video Modeling to Increase Eye Contact. Focus Autism Other Dev. Disabil..

[20] Ahmad W.F.W., Zulkharnain N.A.B. (2020). Development of a Mobile Application Using Augmentative and Alternative Communication and Video Modelling for Autistic Children. Glob. Bus. Manag. Res..

[21] Hernández P., Molina A.I., Lacave C., Rusu C., Toledano-González A. (2022). PlanTEA: Supporting Planning and Anticipation for Children with ASD Attending Medical Appointments. Appl. Sci..

[22] Zink A.G., Molina E.C., Diniz M.B., Santos M.T.B.R., Guaré R.O. (2018). Communication application for use during the first dental visit for children and adolescents with autism spectrum disorders. Pediatr. Dent..

[23] Allen K.D., Vatland C., Bowen S.L., Burke R.V. (2015). An evaluation of parent-produced video self-modeling to improve independence in an adolescent with intellectual developmental disorder and an autism spectrum disorder: A controlled case study. Behav. Modif..

[24] Babb S., Gormley J., McNaughton D., Light J. (2019). Enhancing independent participation within vocational activities for an adolescent with ASD using AAC video visual scene displays. J. Spec. Educ. Technol..

[25] Skillen K.L., Donnelly M.P., Nugent C.D., Booth N. (2016). LifePal: A mobile self-management tool for supporting young people with autism. Proceedings of the XIV Mediterranean Conference on Medical and Biological Engineering and Computing 2016: MEDICON 2016.

[26] Fage C., Consel C., Etchegoyhen K., Amestoy A., Bouvard M., Mazon C., Sauzéon H. (2019). An emotion regulation app for school inclusion of children with ASD: Design principles and evaluation. Comput. Educ..

[27] Bellon-Harn M., Manachaiah V. (2021). Functionality, Impact, and Satisfaction of a Web-Based and Mobile Application Support Program for Students with Autism Spectrum Disorder. Online Learn..

[28] Scheibel G., Zimmerman K.N., Wills H.P. (2022). Increasing On-Task Behavior Using Technology-Based Self-Monitoring: A Meta-Analysis of I-Connect. J. Spec. Educ. Technol..

[29] Hijab M.H.F., Al-Thani D., Banire B. (2021). A Multimodal Messaging App (MAAN) for Adults With Autism Spectrum Disorder: Mixed Methods Evaluation Study. JMIR Form. Res..

[30] Schmidt M., Schmidt C., Glaser N., Beck D., Lim M., Palmer H. (2021). Evaluation of a spherical video-based virtual reality intervention designed to teach adaptive skills for adults with autism: A preliminary report. Interact. Learn. Environ..

[31] Kim B., Lee D., Min A., Paik S., Frey G., Bellini S., Han K., Shih P.C. (2020). PuzzleWalk: A theory-driven iterative design inquiry of a mobile game for promoting physical activity in adults with autism spectrum disorder. PLoS ONE.

[32] Lee D., Frey G.C., Min A., Kim B., Cothran D.J., Bellini S., Han K., Shih P.C. (2020). Usability inquiry of a gamified behavior change app for increasing physical activity and reducing sedentary behavior in adults with and without autism spectrum disorder. Health Inform. J..

[33] Lee D., Frey G.C., Cothran D.J., Harezlak J., Shih P.C. (2022). Effects of a Gamified, Behavior Change Technique–Based Mobile App on Increasing Physical Activity and Reducing Anxiety in Adults With Autism Spectrum Disorder: Feasibility Randomized Controlled Trial. JMIR Form. Res..

[34] Lee T., Kim S., Kim J., Park K.J., Kim H.W. (2022). Efficacy of Mobile-Based Cognitive Training Program DoBrain in Preschool Children With or Without Developmental Disabilities: A Randomized, Single-Blind, Active-Controlled Trial. Psychiatry Investig..

[35] Senette C., Buzzi M.C., Buzzi M., Trujillo A. (2021). Visual Aids for Teaching Piano to Students with Autism: Designing a Web App Through Practice. Proceedings of the Technology-Enhanced Learning for a Free, Safe, and Sustainable World: 16th European Conference on Technology Enhanced Learning, EC-TEL 2021.

[36] Gomez J., Jaccheri L., Torrado J.C., Montoro G. Leo con lula, introducing global reading methods to children with ASD. Proceedings of the 17th ACM Conference on Interaction Design and Children.

[37] Zaffke A., Jain N., Johnson N., Alam M.A.U., Magiera M., Ahamed S.I. (2015). iCanLearn: A mobile application for creating flashcards and social Stories™ for Children with Autism. Proceedings of the Smart Homes and Health Telematics: 12th International Conference, ICOST 2014.

[38] Gallardo-Montes C.d.P., Caurcel Cara M.J., Crisol Moya E., Jarque Fernández S. (2021). Assessment of apps aimed at developing basic instrumental skills in autistic children and teenagers. Mathematics.

[39] Gallardo-Montes C.d.P., Caurcel Cara M.J., Rodríguez Fuentes A. (2022). Technologies in the education of children and teenagers with autism: Evaluation and classification of apps by work areas. Educ. Inf. Technol..

[40] Mykyta A.D., Zhou Z. (2017). Accessing quality apps to promote basic relational concepts acquisition among young children with autism. Psychol. Sch..

[41] Bonnot O., Adrien V., Venelle V., Bonneau D., Gollier-Briant F., Mouchabac S. (2021). Mobile App for Parental Empowerment for Caregivers of Children with Autism Spectrum Disorders: Prospective Open Trial. JMIR Ment. Health.

[42] Préfontaine I., Lanovaz M.J., McDuff E., McHugh C., Cook J.L. (2019). Using mobile technology to reduce engagement in stereotypy: A validation of decision-making algorithms. Behav. Modif..

[43] Law G.C., Neihart M., Dutt A. (2018). The use of behavior modeling training in a mobile app parent training program to improve functional communication of young children with autism spectrum disorder. Autism.

[44] Fuller-Tyszkiewicz M., Richardson B., Little K., Teague S., Hartley-Clark L., Capic T., Khor S., Cummins R.A., Olsson C.A., Hutchinson D. (2020). Efficacy of a smartphone app intervention for reducing caregiver stress: Randomized controlled trial. JMIR Ment. Health.

[45] Siyam N., Abdallah S. (2022). Toward automatic motivator selection for autism behavior intervention therapy. Univers. Access Inf. Soc..

[46] Law G.C., Dutt A., Neihart M. (2019). Increasing intervention fidelity among special education teachers for autism intervention: A pilot study of utilizing a mobile-app-enabled training program. Res. Autism Spectr. Disord..

[47] Bangerter A., Manyakov N.V., Lewin D., Boice M., Skalkin A., Jagannatha S., Chatterjee M., Dawson G., Goodwin M.S., Hendren R. (2019). Caregiver daily reporting of symptoms in autism spectrum disorder: Observational study using web and mobile apps. JMIR Ment. Health.

[48] Barbaro J., Yaari M. (2020). Study protocol for an evaluation of ASDetect—A Mobile application for the early detection of autism. BMC Pediatr..

[49] Chi N.A., Washington P., Kline A., Husic A., Hou C., He C., Dunlap K., Wall D.P. (2022). Classifying Autism From Crowdsourced Semistructured Speech Recordings: Machine Learning Model Comparison Study. JMIR Pediatr. Parent..

[50] Kalantarian H., Jedoui K., Dunlap K., Schwartz J., Washington P., Husic A., Tariq Q., Ning M., Kline A., Wall D.P. (2020). The performance of emotion classifiers for children with parent-reported autism: Quantitative feasibility study. JMIR Ment. Health.

[51] Varma M., Washington P., Chrisman B., Kline A., Leblanc E., Paskov K., Stockham N., Jung J.Y., Sun M.W., Wall D.P. (2022). Identification of Social Engagement Indicators Associated with Autism Spectrum Disorder Using a Game-Based Mobile App: Comparative Study of Gaze Fixation and Visual Scanning Methods. J. Med. Internet Res..

[52] Egger H.L., Dawson G., Hashemi J., Carpenter K.L., Espinosa S., Campbell K., Brotkin S., Schaich-Borg J., Qiu Q., Tepper M. (2018). Automatic emotion and attention analysis of young children at home: A ResearchKit autism feasibility study. NPJ Digit. Med..

[53] Barbaro J., Dissanayake C., Sadka N. Universal developmental surveillance for autism in infants, toddlers and preschoolers: The Social Attention and Communication Study-Revised (SACS-R) and SACS-Preschool. Proceedings of the Annual Meeting of the International Society for Autism Research.

[54] Thabtah F. (2019). An accessible and efficient autism screening method for behavioural data and predictive analyses. Health Inform. J..

[55] Feller C., Ilen L., Eliez S., Schneider M. (2023). Characterizing daily-life social interactions in adolescents and young adults with neurodevelopmental disorders: A comparison between individuals with Autism Spectrum Disorders and 22q11. 2 deletion syndrome. J. Autism Dev. Disord..

[56] Davis M., Dautenhahn K., Nehaniv C., Powell S. (2006). Towards an interactive system eliciting narrative comprehension in children with autism: A longitudinal study. Designing Accessible Technology.

[57] Bondy A.S., Frost L.A. (1994). The picture exchange communication system. Focus Autistic Behav..

[58] Schlosser R.W., Wendt O. (2008). Effects of augmentative and alternative communication intervention on speech production in children with autism: A systematic review. Am. J. Speech-Lang. Pathol..

[59] Charlop-Christy M.H., Le L., Freeman K.A. (2000). A comparison of video modeling with in vivo modeling for teaching children with autism. J. Autism Dev. Disord..

[60] Michie S., Johnston M., Carey R. (2020). Behavior change techniques. Encyclopedia of Behavioral Medicine.

[61] Dobson K.S., McEpplan A.M., Dobson D. (2019). Empirical Validation and the Cognitive-Behavioral Therapies.

[62] Rotheram-Fuller E., MacMullen L. (2011). Cognitive-behavioral therapy for children with autism spectrum disorders. Psychol. Sch..

[63] Taylor P.J., Russ-Eft D.F., Chan D.W. (2005). A meta-analytic review of behavior modeling training. J. Appl. Psychol..

[64] Konstantareas M.M., Stewart K. (2006). Affect regulation and temperament in children with autism spectrum disorder. J. Autism Dev. Disord..

[65] Boehm A.E. (2011). Boehm Test of Basic Concepts-3.

[66] Zhao W., Liu X., Qiu T., Luo X. (2020). Virtual avatar-based life coaching for children with autism spectrum disorder. Computer.

[67] Matallaoui A., Hanner N., Zarnekow R., Stieglitz S., Lattemann C., Robra-Bissantz S., Zarnekow R., Brockmann T. (2017). Introduction to Gamification: Foundation and Underlying Theories. Gamification: Using Game Elements in Serious Contexts.

[68] Hunicke R., LeBlanc M., Zubek R. MDA: A formal approach to game design and game research. Proceedings of the AAAI Workshop on Challenges in Game AI.

[69] Groh F. (2012). Gamification: State of the Art Definition and Utilization. RTMI ’12—Proceedings of the 4th Seminar on Research Trends in Media Informatics.

[70] Deci E.L., Ryan R.M. (2013). Intrinsic Motivation and Self-Determination in Human Behavior.

[71] Nakamura J., Csikszentmihalyi M. (2009). Flow theory and research. Handb. Posit. Psychol..

[72] Milgram P., Kishino F. (1994). A taxonomy of mixed reality visual displays. IEICE Trans. Inf. Syst..

[73] Reed S.R., Stahmer A.C., Suhrheinrich J., Schreibman L. (2013). Stimulus overselectivity in typical development: Implications for teaching children with autism. J. Autism Dev. Disord..

[74] Vredenburg K., Mao J.Y., Smith P.W., Carey T. A survey of user-centered design practice. Proceedings of the SIGCHI Conference on Human Factors in Computing Systems.

[75] Aguiar Y.P.C., Galy E., Godde A., Trémaud M., Tardif C. (2022). AutismGuide: A usability guidelines to design software solutions for users with autism spectrum disorder. Behav. Inf. Technol..

[76] Beukelman D.R., Mirenda P., Paul H. (1998). Augmentative and Alternative Communication.

[77] Mirenda P. (2003). Toward functional augmentative and alternative communication for students with autism. Lang. Speecch Hear. Serv. Sch..

[78] Bellini S., Akullian J. (2007). A meta-analysis of video modeling and video self-modeling interventions for children and adolescents with autism spectrum disorders. Except. Child..

[79] Bandura A., Walters R.H. (1977). Social Learning Theory.

[80] Davis R., Campbell R., Hildon Z., Hobbs L., Michie S. (2015). Theories of behaviour and behaviour change across the social and behavioural sciences: A scoping review. Health Psychol. Rev..

[81] Decker P.J., Nathan B.R. (1985). Behavior Modeling Training: Principles and Applications.

[82] Bennett C.L., Brady E., Branham S.M. Interdependence as a frame for assistive technology research and design. Proceedings of the 20th International ACM Sigaccess Conference on Computers and Accessibility.

